# Regulation of phosphatase and tensin homolog by complement component 5a (C5a) and its receptor (C5aR1) in lupus nephritis: A novel therapeutic target

**DOI:** 10.1002/ccs3.70055

**Published:** 2025-12-19

**Authors:** Yuehong Ma, Yi Wang, Peng Zhao, Li Cheng, Lei Li, Rongshan Li, Xiaoshuang Zhou

**Affiliations:** ^1^ Shanxi Provincial Key Laboratory of Kidney Disease Taiyuan China; ^2^ Department of Nephrology Shanxi Provincial People's Hospital The Fifth Clinical Medical College of Shanxi Medical University Taiyuan China; ^3^ Shanxi Medical University Taiyuan China; ^4^ Department of Rheumatology and Immunology Shanxi Provincial People's Hospital The Fifth Clinical Medical College of Shanxi Medical University Taiyuan China; ^5^ Department of Dermatology Shanxi Provincial People's Hospital The Fifth Clinical Medical College of Shanxi Medical University Taiyuan China; ^6^ Department of General Practice Shanxi Provincial People's Hospital The Fifth Clinical Medical College of Shanxi Medical University Taiyuan China

**Keywords:** complement component 5a, complement component 5a receptor, lupus nephritis, phosphatase and tensin homolog, phosphoinositide 3‐kinase/AKT serine/threonine kinase pathway

## Abstract

Lupus nephritis (LN), a renal manifestation of systemic lupus erythematosus, results from immune‐mediated kidney injury. The present study investigated how complement component 5a (C5a) and its receptor (C5aR1) regulate phosphatase and tensin homolog (PTEN) expression and the phosphoinositide 3‐kinase (PI3K)/AKT pathway during LN development. Using MRL/lpr mice as an LN model, we examined the expression of C5a, C5aR1, PTEN, and related proteins through Western blot, quantitative real‐time PCR, and immunohistochemistry. Treatment with a C5aR1 antagonist (C5aR1A) was administered to assess its effects on renal function and molecular parameters. Elevated expression of C5a and C5aR1 was detected in MRL/lpr mice, accompanied by reduced PTEN levels and enhanced PI3K/AKT signaling activity. Treatment with the C5aR1 antagonist (C5aR1A) restored PTEN expression, suppressed AKT phosphorylation, and improved renal function, reflected by lower serum creatinine and blood urea nitrogen concentrations. These findings suggest that the C5a/C5aR1 axis contributes to LN progression by regulating PTEN and the PI3K/AKT signaling pathway, offering potential therapeutic insights for LN treatment.

## Graphical abstract

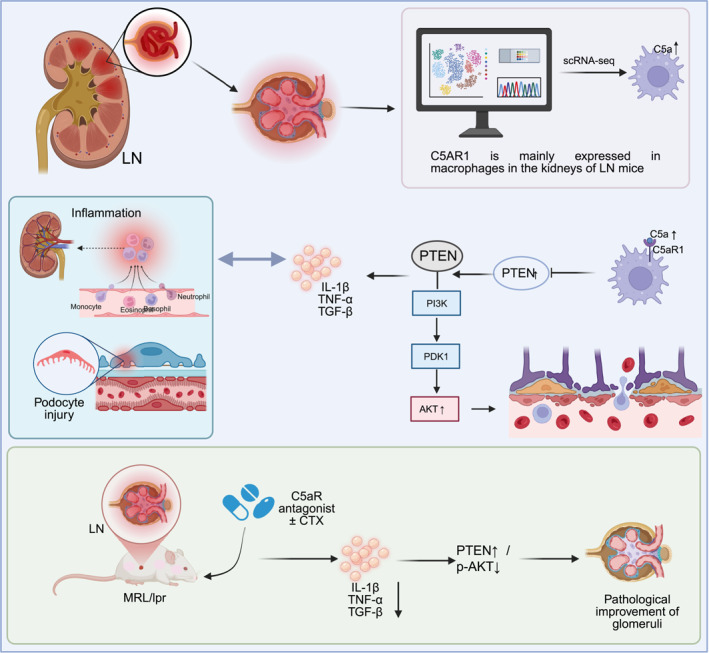

## 1 INTRODUCTION

Systemic lupus erythematosus (SLE) represents a multifactorial autoimmune disorder affecting multiple organ systems.^1^ Its pathogenesis involves impaired immune tolerance, immune complex accumulation, and excessive activation of pro‐inflammatory mediators.^2^, ^3^ Among its clinical manifestations, lupus nephritis (LN) is one of the most common and severe forms of organ damage, significantly impairing patients' quality of life and long‐term prognosis.^4^, ^5^ The pathological features of LN include glomerular immune complex deposition, infiltration of inflammatory cells, and structural tissue damage.^5^ Clinically, LN presents with diverse manifestations, often including proteinuria, hematuria, hypertension, and abnormalities in renal function.^6^ Although glucocorticoids and immunosuppressants are widely used to control disease activity, their therapeutic efficacy varies considerably among individuals, and a subset of patients still progresses to end‐stage renal disease.^7^ Considering the prevalence and complexity of LN, elucidating its molecular basis, especially the contribution of inflammatory signaling networks to disease progression, is crucial for advancing targeted therapeutic development.

Aberrant activation of the complement system has been widely recognized as a major pathogenic factor in the development of LN, with increasing attention focused on the terminal complement component 5a (C5a) and complement component 5a receptor (C5aR1) in recent years.^8^ C5a is a potent chemoattractant and proinflammatory mediator that induces the migration of neutrophils and monocytes, activates endothelial cells, and promotes the release of inflammatory cytokines, ultimately exacerbating renal inflammatory injury.^9^, ^10^ C5aR1, the principal signal transduction receptor for C5a, is upregulated in various immune‐related kidney disease models, where its activation closely correlates with the extent of tissue damage.^11^ In both LN patients and animal models, elevated expression of C5a and C5aR1 has been positively associated with disease activity and renal injury markers,^12^ implying a key regulatory function of this axis in LN pathogenesis.^13^ However, whether C5a/C5aR1 activity extends beyond extracellular inflammatory responses to modulate intracellular signaling remains to be clarified.

The phosphoinositide 3‐kinase (PI3K)/AKT signaling cascade is vital for controlling cellular growth, metabolism, apoptosis, and inflammatory regulation. Sustained activation of this pathway has been documented in multiple renal disorders and closely correlates with tissue injury and inflammation.^14^, ^15^ Phosphatase and tensin homolog (PTEN), a key negative regulator of this pathway, dephosphorylates phosphatidylinositol (3,4,5)‐trisphosphate (PIP3), thereby inhibiting AKT phosphorylation and activation.^16^, ^17^ Studies have shown that in models of chronic kidney disease and immune‐mediated nephropathies, PTEN downregulation is often accompanied by excessive activation of the PI3K/AKT pathway, thereby promoting inflammatory responses and cellular injury.^18^ Although the regulatory relationship between PI3K/AKT and PTEN has been extensively studied, direct evidence for whether this pathway functions as a downstream target of the C5a/C5aR1 axis in LN pathogenesis remains lacking. Whether the complement system contributes to AKT activation and promotes renal inflammation and tissue injury through modulation of PTEN expression represents a promising new direction for future research.^19^, ^20^

Building on the above background, this study aimed to investigate the role of the C5a/C5aR1‐PTEN/PI3K/AKT regulatory axis in the pathogenesis of LN. Using MRL/lpr mice as an LN model, we examined the expression and interaction of C5a, C5aR1, PTEN, and related signaling proteins, and evaluated the effects of a C5aR1 antagonist on renal injury and molecular changes. By elucidating how C5a/C5aR1 signaling modulates PTEN and AKT activation, this study aims to clarify the molecular mechanisms linking complement activation to intracellular signaling pathways in LN and to provide experimental evidence to support the development of more targeted therapeutic approaches.

## 2 MATERIALS AND METHODS

### 2.1 scRNA‐seq analysis

Single‐cell RNA sequencing (scRNA‐seq) data (GSE279823) from renal tissues of SLE mice were obtained from the Gene Expression Omnibus (GEO) database and analyzed using Seurat (R package). After standard quality control (nFeature_RNA > 200, nCount_RNA < 20,000, percent.mt < 4), the 2000 most variable genes were selected for principal component analysis (PCA). The first 16 principal components were selected for clustering (resolution = 0.5), followed by uniform manifold approximation and projection (UMAP) visualization. Cell types were identified based on canonical markers and the CellMarker database. Differentially expressed genes (DEGs) were identified using the Wilcoxon rank‐sum test implemented in Seurat.

### 2.2 GEO dataset analysis

SLE‐related renal tissue datasets were retrieved from the GEO database (https://www.ncbi.nlm.nih.gov/geo/). The dataset GSE32591, based on the GPL14663 platform, included 46 glomerular samples (14 from control subjects and 32 from SLE patients) and was analyzed. GSE112943, based on the GPL10558 platform, comprised 21 renal tissue samples (7 controls and 14 SLE patients). GSE160488, based on the GPL21810 platform, contained sequencing data from renal tissues of 3 MRL/MpJ mice (controls) and 3 MRL/lpr mice (SLE model). DEGs were determined using the R package limma, with control samples as references. Genes with |log_2_FC| > 0 and *p <* 0.05 were considered significant, and multiple testing correction was performed using the Benjamini–Hochberg method. The expression of C5AR1 across datasets was visualized using the R package ggplot2.

### 2.3 Animal experiments

A total of 25 female mice were used in this study, including 20 MRL/lpr mice with LN and 5 age‐matched C57BL/6J mice (6–7 weeks old, 20 ± 1.5 g). C57BL/6J mice were obtained from the Experimental Animal Center of Shanxi Provincial People's Hospital, and MRL/lpr mice were purchased from the Model Animal Research Center of Nanjing University (originally derived from The Jackson Laboratory). Animals were housed under specific pathogen‐free conditions (24 ± 2°C, 40%–70% humidity, 12‐h light/dark cycle) with ad libitum access to food and water. All experimental procedures were approved by the Institutional Animal Care and Use Committee of Shanxi Provincial People's Hospital (approval no. 2021136).

Mice were randomly allocated into five groups (*n* = 5 each): wild‐type control (WT), LN model, C5aR antagonist (LN + C5aRA1), combined treatment (LN + C5aRA1 + CTX), and positive control (LN + CTX). WT and LN groups received an equal volume of saline by oral gavage for 12 weeks. The LN + C5aRA1 group received PMX53, a C5aR1 antagonist (10 mg/kg; HY‐106178, MedChemExpress), once daily by oral gavage. The LN + C5aRA1 + CTX group received PMX53 (10 mg/kg) and cyclophosphamide (CTX, 20 mg/kg/day), whereas the LN + CTX group was treated with CTX alone (20 mg/kg/day) for 12 weeks.

Twelve hours after the final administration, mice were euthanized, and blood samples were collected. Serum was obtained by centrifugation at 2096 × g for 15 min at 4°C and stored at −20°C. Serum creatinine (Scr) and blood urea nitrogen (BUN) were measured using an automatic biochemical analyzer. Kidney tissues were fixed in 10% neutral buffered formalin for histological analysis, and the remaining tissues were frozen in liquid nitrogen and stored at −80°C for molecular assays. Cytokine levels in kidney homogenates were analyzed using a Mouse Cytokine Antibody Array, Panel A (R&D Systems, #ARY006). Each group consisted of five biological replicates (*n* = 5).

### 2.4 Cell culture

Human mesangial cells were provided by the Key Laboratory of Nephrology, Shanxi Province. Cells were maintained in Dulbecco's Modified Eagle Medium enriched with 10% fetal bovine serum, 100 U/mL penicillin, and 100 μg/mL streptomycin (37°C, 5% CO_2_).

### 2.5 Mesangial cell experiments

C5aR1 gene silencing was achieved using small interfering RNA (siRNA), purchased from HAM Bio. For in vitro experiments, specific siRNA targeting C5aR1 (siC5aR1) or a negative control siRNA (siNC) was transfected into glomerular mesangial cells using LipoFiterTM 3.0 liposomal transfection reagent (HAM Bio). For in vivo experiments, a short hairpin RNA (shRNA) targeting C5aR1 (sense strand: 5′‐GCUUUCUGCUGGUGUUUAAdTdT‐3′) was designed and cloned into the pLKO.1 lentiviral vector. Recombinant plasmids and packaging vectors (psPAX2 and pMD2.G) were co‐transfected into HEK293T cells, and high‐titer lentivirus (>1 × 10^8^ TU/mL) was produced after ultracentrifugation. Under direct visualization, 50 μL of viral suspension (3 × 10^8^ TU/mL) was locally injected into the renal parenchyma of LN model mice (dose: 50 μL/20 g body weight). Kidney tissues were collected 7 days after injection, and Western blot (WB) analysis confirmed that C5aR1 protein expression was reduced by more than 70% (*p <* 0.01, *n* = 5). Mice in the negative control group received a non‐targeting shRNA virus (shNC). The shRNA sequence was as follows: Forward (F), 5′‐GCUUUCUGCUGGUGUUUAAdTdT‐3′; Reverse (R), 5′‐UUAAACACCAGCAGAAAGCdTdT‐3′.

### 2.6 Phosphorylation protein array analysis

The Human/Mouse AKT Pathway Phosphorylation Array C1 (Catalog #AAH‐AKT‐1‐2, RayBiotech) was used to detect 18 phosphorylated proteins in human and mouse samples. Three representative targets identified from the array were selected for presentation and subsequent validation in this study.

### 2.7 WB analysis

Glomerular mesangial cells were detached with trypsin and lysed together with kidney tissue homogenates using radioimmunoprecipitation assay buffer to extract total protein. Protein samples were separated by 10% SDS‐polyacrylamide gel electrophoresis and transferred onto polyvinylidene difluoride membranes (Millipore). After blocking, membranes were incubated overnight at 4°C with primary rabbit antibodies, including C5aR (1:500, Catalog #21316‐1‐AP, Proteintech), PTEN (Catalog #9559, Cell Signaling Technology), phosphorylated‐AKT (p‐AKT, Ser473; Catalog #4060, Cell Signaling Technology), AKT (Catalog #9272, Cell Signaling Technology), and GAPDH (Catalog #2118, Cell Signaling Technology). After washing, membranes were exposed to horseradish peroxidase‐conjugated secondary antibodies. Protein signals were visualized using an enhanced chemiluminescence kit (Millipore) and imaged with the Quantity One system (Bio‐Rad). Band intensities were quantified using ImageJ, normalized to β‐actin, and expressed relative to control levels (set as 1). For cytokine arrays, grayscale signal intensities were analyzed and reported as relative pixel density values.

### 2.8 qRT‐PCR analysis

Total RNA was extracted using TRIzol reagent (15596026CN, Invitrogen™, Thermo Fisher) and quantified with a Biotek microplate reader (Agilent Technologies). Reverse transcription was performed using 500 ng of RNA with the PrimeScript™ RT Master Mix (Takara). Quantitative real‐time PCR (qRT‐PCR) was performed with TB Green® Premix Ex Taq™ II (Tli RNaseH Plus, Takara). The primer sequences used were as follows:

IL‐1β: Forward (F): 5′‐TGACC TGGGC TGTCC TGATG‐3′; Reverse (R): 5′‐GGTGC TCATG TCCCT CATCC TG‐3′.
TNF‐α: Forward (F): 5′‐GTCGT AGCAA ACCAC CAAG‐3′; Reverse (R): 5′‐GTCGC CTCAC AGAGC AAT‐3′.
β‐actin (internal control gene): Forward (F): 5′‐ACCTT CTACA ATGAG CTGCG‐3′; Reverse (R): 5′‐CCTGG ATAGC AACGT ACATG G‐3′.

Relative mRNA expression was calculated using the 2^−ΔΔCt^ method and normalized to β‐actin expression.

### 2.9 Renal histopathological preparation

Kidney tissues were harvested following in situ perfusion of anesthetized mice and fixed in 4% paraformaldehyde. Fixed samples were paraffin‐embedded and sectioned at 4 μm for histopathological examination. Hematoxylin and eosin (H&E) and periodic acid–Schiff (PAS) staining were performed to assess mesangial proliferation and general renal pathology. For immunohistochemistry, kidney samples were fixed in 4% paraformaldehyde for 24 h, dehydrated, and embedded in paraffin. Sections (4 μm) were mounted on poly‐L‐lysine‐coated glass slides and baked at 60°C for 1 h to enhance adhesion, then deparaffinized in xylene and rehydrated through graded ethanol. Antigen retrieval was performed by heat‐induced epitope retrieval, and nonspecific binding was blocked with normal goat serum. Sections were incubated overnight at 4°C with rabbit primary antibodies, including C5aR1 (Proteintech), IL‐1β (Proteintech, Cat#16806‐1‐AP), monocyte chemoattractant protein‐1 (MCP‐1) (Abcam, Cat#ab25124), TNF‐α (Abcam, Cat#ab6671), and transforming growth factor‐β (TGF‐β) (Proteintech, Cat#21898‐1‐AP). The next day, sections were brought to 37°C for 30 min, washed in phosphate‐buffered saline (PBS), incubated with secondary antibodies for 10 min at ambient temperature, and treated with streptavidin–peroxidase for 10 min. After additional PBS washes, staining was developed using 3,3′‐Diaminobenzidine chromogen and counterstained with hematoxylin. Positive signals were observed under a light microscope, and PAS staining was quantified as the percentage of positive area using Image‐Pro Plus software.^21^

### 2.10 Statistical analysis

All data were analyzed using SPSS version 26.0. Quantitative variables were expressed as mean ± standard deviation (mean ± SD). Group comparisons were conducted using two‐tailed independent‐sample *t*‐tests or one‐way analysis of variance, followed by least significant difference (LSD) or Tukey's post hoc tests to determine intergroup differences. For data not following a normal distribution, nonparametric analyses were applied, including the Mann–Whitney *U* test or Kruskal–Wallis test. Differences in protein or mRNA expression levels from experiments such as WB, qRT‐PCR, and immunohistochemistry were quantified using image analysis software and statistically evaluated. A *p*‐value <0.05 was considered statistically significant.

## 3 RESULTS

### 3.1 C5aR11 was primarily expressed in renal macrophages of SLE mice

To assess C5aR1 expression in SLE‐associated renal tissues, we analyzed the scRNA‐seq dataset GSE279823 from the GEO database. Quality control was based on gene count (nFeature_RNA), transcript count (nCount_RNA), and mitochondrial gene percentage (percent.mt), applying thresholds of nFeature_RNA > 200, nCount_RNA < 20,000, and percent.mt < 4. Post‐filtering metrics, including nFeature_RNA, nCount_RNA, percent.mt, percent.rb, and percent.HB, are shown in Figure S1A,B. Correlation analysis revealed a negative relationship between nCount_RNA and percent.mt (*r* = −0.22) and a strong positive correlation between nCount_RNA and nFeature_RNA (*r* = 0.96), confirming high data quality for downstream analyses (Figure S1C).

Highly variable genes were identified using the FindVariableFeatures function, with the top 2000 genes selected based on variance (Figure S1D). These genes were subjected to PCA for linear dimensionality reduction. The top gene loadings and corresponding heatmaps for PC_1–PC_4 were visualized (Figure S1E), along with the two‐dimensional cell distribution across PC_1 and PC_2 (Figure S1F). The JackStrawPlot function was used to assess the *p*‐value distribution of PC_1–PC_20 relative to a uniform reference (Figure S1G), whereas the Elbow Plot showed the variance explained by each component (Figure S1H). Collectively, PC_1–PC_16 accounted for most of the variance among highly variable genes, representing the optimal range for downstream analysis (Figures S1G,H).

**FIGURE 1:**
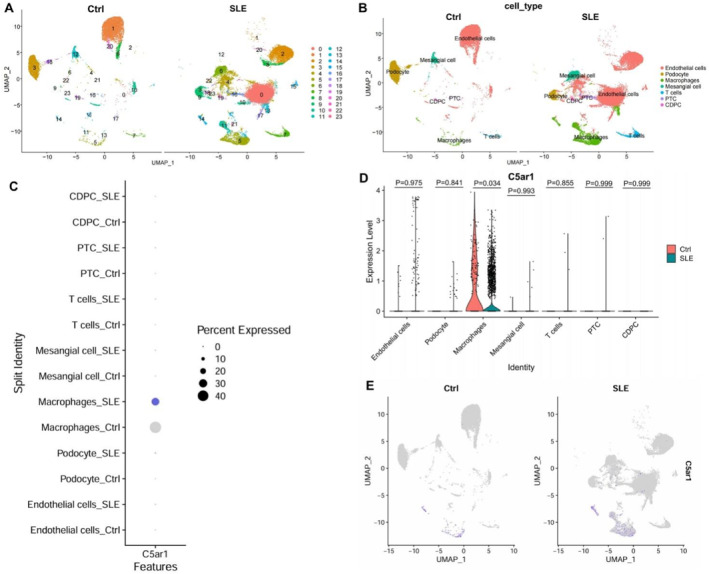
Expression distribution of C5aR11 in the single‐cell dataset GSE279823. (A) UMAP clustering results (resolution = 0.5) from dataset GSE279823, shown separately for the control and SLE groups; (B) UMAP plot of cell type annotations, shown separately for the control and SLE groups; (C–E) Expression and distribution of C5aR11 across various cell types. Statistical testing was performed using the Wilcoxon rank‐sum test implemented in the Seurat package. *p >* 0.05, not significant. UMAP, uniform manifold approximation and projection; SLE, systemic lupus erythematosus.

To further characterize renal cell populations, nonlinear dimensionality reduction was performed on PC_1–PC_16 using the UMAP algorithm. At a resolution of 0.5 (Figure S2A), 24 distinct clusters were identified (Figure 1A), and their marker gene expression profiles were visualized in a heatmap (Figure S2B). Cell type annotation was conducted based on signature gene expression patterns combined with the CellMarker 2.0 database (http://bio‐bigdata.hrbmu.edu.cn/CellMarker/index.html), resulting in seven major cell types: endothelial cells (Emcn, Egfl7, Ly6c1), podocytes (Nphs2, Ptpro, Synpo), macrophages (Cd68, Cd74, Il1b), mesangial cells (Pdgfrb, Gata3, Itga8), T cells (Cd3g, Cd3d, Cd3e), proximal tubular cells (Lrp2, Slc34a1, Acsm2), and collecting duct principal cells (Aqp2, Aqp3, Fxyd4) (Figure 1B; Figure S2C,D). Specifically, clusters 0, 1, 2, 4, 8, 10, 15, 17, 20, and 22 were annotated as endothelial cells; clusters 3, 9, 18, and 23 as podocytes; clusters 5, 11, 13, 14, and 21 as macrophages; clusters 6 and 12 as mesangial cells; cluster 7 as T cells; cluster 16 as PTCs; and cluster 19 as CDPCs. The marker genes and their expression patterns for each cell type are shown in Figure S2C.

We then analyzed the distribution of C5aR11 within SLE‐related renal tissues. The results revealed that C5aR11 was predominantly expressed in macrophages and was markedly upregulated in SLE samples relative to healthy counterparts (Figure 1C–E).

These findings indicate that C5aR11 is primarily expressed in macrophages within SLE‐associated renal tissues and is more abundant in SLE mice than in controls.

### 3.2 C5aR11 expression was significantly upregulated in SLE renal tissue

**FIGURE 2:**
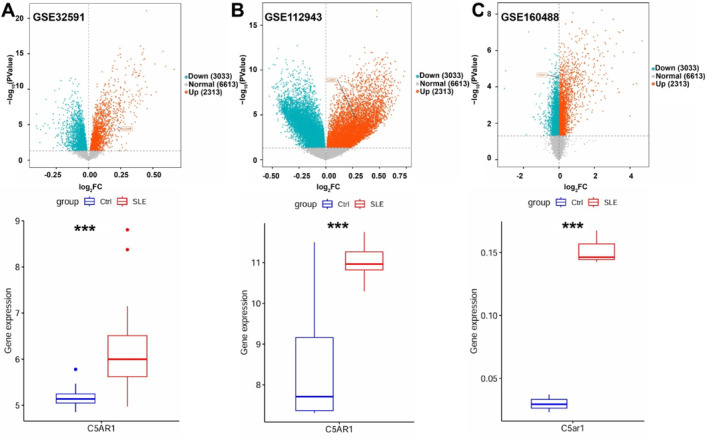
RNA‐seq reveals significant upregulation of C5aR11 in SLE renal tissue. (A–C) Expression levels of C5aR11 in datasets GSE32591 (human; n.control = 14, n.SLE = 32), GSE112943 (human; n.control = 7, n.SLE = 14), and GSE160488 (mouse; n.control = 3, n.SLE = 3), shown as volcano plots (top) and box plots (bottom). DEGs were identified using the criteria |log_2_FC| > 0 and *p <* 0.05, with FDR correction performed using the Benjamini‐Hochberg method. *p >* 0.05, not significant. ***p < 0.001. DEGs, differentially expressed genes; FDR, false discovery rate; SLE, systemic lupus erythematosus.

To further validate the expression of C5aR11 in SLE renal tissues, we analyzed transcriptomic datasets related to SLE kidneys obtained from the GEO database. The findings indicated that C5aR11 expression was significantly elevated in the SLE group compared to controls in all three datasets: GSE32591 (Log_2_FC = 0.2131; *p* = 0.0000133), GSE112943 (Log_2_FC = 0.2905; *p* = 0.0003323), and GSE160488 (Log_2_FC = 0.1595; *p* = 0.000033) (Figure 2A–C).

These findings suggest that elevated C5aR11 expression is closely associated with SLE‐related renal injury (Figure 2).

### 3.3 C5a promoted inflammatory cytokine expression and activated renal inflammation in lupus mice

**FIGURE 3:**
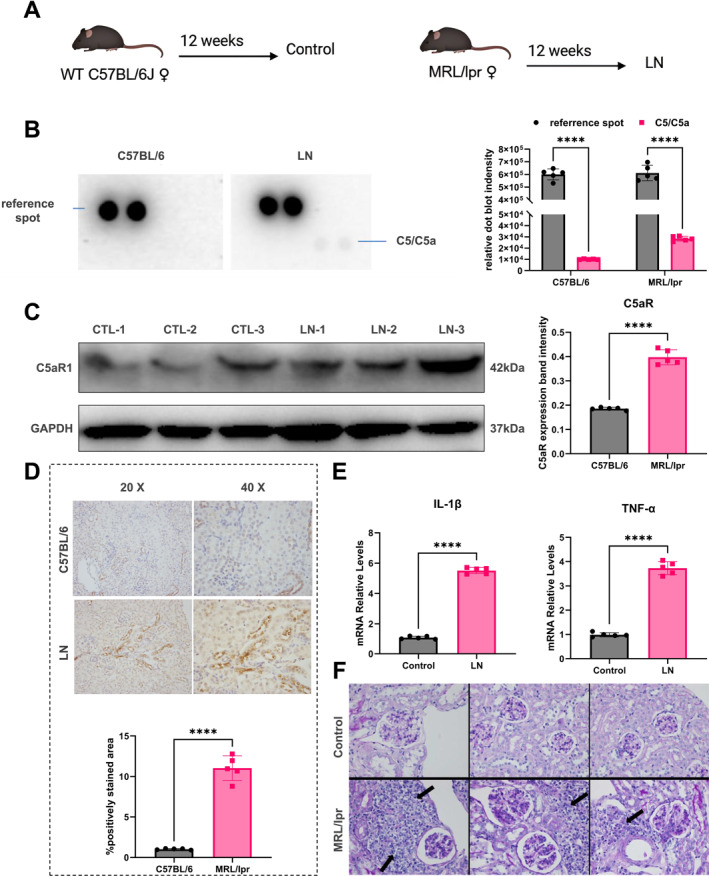
Upregulation of C5a/C5aR1 promotes cytokine release and drives inflammation in LN mice. (A) Schematic diagram of the animal model induction process; (B) Mouse cytokine antibody array Panel (A) showing increased C5a expression; (C) Western blot analysis of C5aR1 expression in mouse renal tissues, with β‐actin as the internal control (*n* = 5); (D) Immunohistochemical analysis of C5aR1 expression in paraffin‐embedded kidney sections (*n* = 5); (E) Analysis of IL‐1β and TNF‐α mRNA expression in mouse kidney tissues, with β‐actin as the internal control (*n* = 5). (F) Representative H&E staining of paraffin‐embedded kidney sections (*n* = 5); black arrows indicate inflammatory cell infiltration around renal tubules. Group comparisons were performed using a two‐tailed unpaired *t*‐test, followed by LSD or Tukey post hoc tests to determine intergroup differences. Immunohistochemistry scores were analyzed using nonparametric tests. *p >* 0.05, not significant. ****p < 0.0001. H&E, hematoxylin and eosin; LN, lupus nephritis; LSD, least significant difference.

We established an LN mouse model using MRL/lpr mice (Figure 3A). Time‐dependent experiments revealed a marked increase in C5/C5a protein levels in renal tissues of MRL/lpr mice (Figure 3B). Similarly, C5aR1 protein expression was also significantly upregulated in the kidneys of these mice (Figure 3C,D). In the MRL/lpr LN model, C5a activation enhanced inflammatory cell activity and stimulated the release of pro‐inflammatory cytokines such as IL‐1β and TNF‐α (Figure 3E). These results indicate that C5a may promote inflammatory lesion development in MRL/lpr mice by upregulating IL‐1β and TNF‐α expression. In addition, H&E staining revealed pronounced pathological changes in the MRL/lpr group compared to controls, including diffuse mesangial cell proliferation and matrix expansion in glomeruli, the presence of occasional cellular crescents within Bowman's capsule, widespread degeneration of tubular epithelial cells with vacuolization and luminal dilation, as well as protein casts in some areas. Thickened arteriolar walls and perivascular inflammatory cell infiltration were also observed (Figure 3F). These findings prompted the design of additional in vivo experiments to investigate the mechanism by which C5a drives the synthesis of pro‐inflammatory cytokines in MRL/lpr mice.

### 3.4 C5aR1A alleviated renal inflammation and injury in LN mice

**FIGURE 4:**
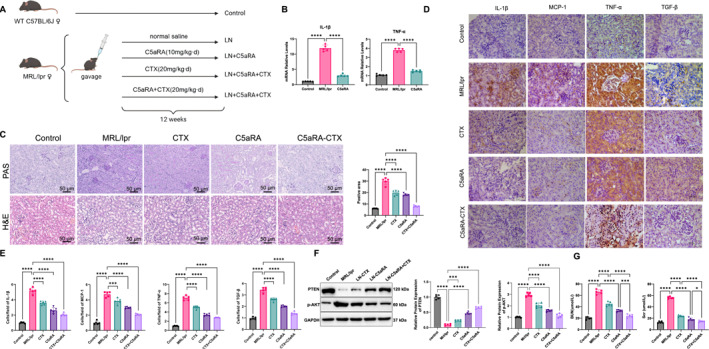
C5aR1A alleviates inflammation and renal dysfunction in LN mice. (A) Schematic diagram of the animal model procedure; (B) mRNA expression levels of IL‐1β and TNF‐α in mouse renal tissue, normalized to β‐actin (*n* = 5); (C) PAS and H&E staining of paraffin‐embedded kidney sections (*n* = 5); (D, E) Immunohistochemical analysis of IL‐1β, MCP‐1, TNF‐α, and TGF‐β expression in renal tissues at 40× magnification (*n* = 5); (F) Western blot analysis of PTEN and p‐AKT in mouse kidney tissue, with GAPDH as a loading control (*n* = 5); (G) Quantification of BUN and Scr levels in mouse kidney tissues (*n* = 5). Statistical analysis was performed using one‐way ANOVA. *p >* 0.05, not significant. *p < 0.05; ***p < 0.001; ****p < 0.0001. ANOVA, analysis of variance; BUN, blood urea nitrogen; H&E, hematoxylin and eosin; LN, lupus nephritis; MCP‐1, monocyte chemoattractant protein‐1; PAS, periodic acid–Schiff; PTEN, phosphatase and tensin homolog; Scr, serum creatinine; TGF‐β, transforming growth factor‐β.

We subsequently administered a C5aR1A to further investigate its effect on LN progression (Figure 4A). The results demonstrated that C5aR1A treatment significantly reduced inflammation and renal dysfunction in MRL/lpr mice. RT‐qPCR analysis demonstrated that renal IL‐1β and TNF‐α mRNA levels were significantly lower in the C5aR1A‐treated group than in the LN group, with a more pronounced inhibitory effect than that observed with CTX monotherapy (Figure 4B). PAS and H&E staining confirmed that C5aR1A effectively ameliorated LN‐associated pathological damage (Figure 4C). In the LN group, typical pathological features were observed, including mesangial matrix expansion with dark‐purple deposits, distorted PAS‐positive basement membranes, obliterated tubular lumens, and prominent proteinaceous casts. In contrast, the C5aR1A group exhibited substantial histological improvement, characterized by the restoration of glomerular capillary patency and a reduction in interstitial inflammatory infiltration. Immunohistochemistry further revealed that C5aR1A suppressed the expression of key inflammatory mediators (Figure 4D,E). Levels of MCP‐1 and TGF‐β were also significantly reduced following C5aR1A treatment. WB analysis indicated that the therapeutic effect of C5aR1A was mediated through restoration of the PTEN/p‐AKT pathway. Specifically, PTEN protein expression was significantly upregulated in the C5aR1A‐treated group compared with the LN group, showing a stronger effect than CTX treatment. In contrast, p‐AKT levels were decreased, displaying a clear inverse correlation with PTEN restoration (Figure 4F). We measured serum BUN and Scr levels and found that both were significantly decreased following C5aRA and CTX treatment, indicating improved renal function (Figure 4G). Collectively, these findings highlight the pivotal role of the C5a/C5aR1 signaling axis in mediating inflammatory injury in LN and suggest it as a potential therapeutic target.

### 3.5 C5a activates the PI3K/AKT pathway by negatively regulating PTEN activity

**FIGURE 5:**
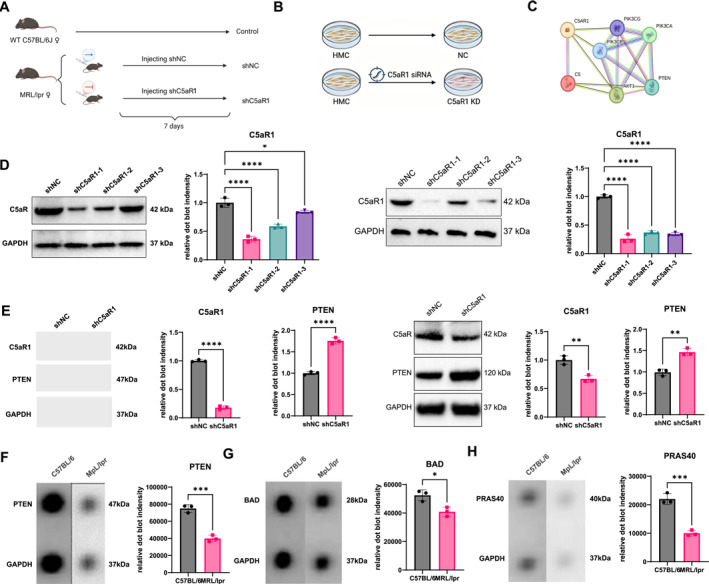
C5a suppresses PTEN expression and enhances AKT pathway activation to promote inflammation. (A) Schematic diagram of the animal model procedure; (B) Schematic diagram of the in vitro cell experiment; (C) Protein‐protein interaction network illustrating key molecules linking C5a/C5aR11 with PTEN and the PI3K/AKT signaling pathway (confidence score = 0.15); (D) Western blot analysis of C5aR1 knockdown by three siRNAs in vitro (D1–D2) and by three shRNAs in kidney tissues in vivo (D3–D4), with GAPDH as loading control (*n* = 3); (E) Western blot analysis of C5aR1 and PTEN expression after C5aR1 knockdown by siRNAs in vitro (E1–E3) and by shRNAs in kidney tissues in vivo (E4–E6), with GAPDH as loading control (*n* = 3). (F–H) Results of the human/mouse AKT pathway phosphorylation antibody array C1 (RayBiotech) showing the expression of BAD, PRAS40, and PTEN. Group comparisons were performed using a two‐tailed unpaired *t*‐test or one‐way ANOVA. *p >* 0.05, not significant; *p < 0.05; **p < 0.01; ***p < 0.001; ****p < 0.0001. ANOVA, analysis of variance; BAD, Bcl‐2‐associated death promoter; PTEN, phosphatase and tensin homolog.

Lentiviral‐mediated gene intervention was performed in mice to assess in vivo effects, accompanied by siRNA transfection experiments in mesangial cells (Figure 5A,B). Analysis revealed that C5a modulated PTEN expression through the PI3K/AKT axis. STRING‐based protein interaction analysis indicated potential associations among C5a/C5aR1, PTEN, and PI3K/AKT signaling components (Figure 5C). Three siRNAs targeting C5aR1 were designed, and WB analysis demonstrated that siC5aR1‐1 and shC5aR1‐1 achieved the highest knockdown efficiency (Figure 5D). Inhibition of C5aR1 activity led to restoration of PTEN expression (Figure 5E). RayBiotech AKT‐array analysis showed significantly reduced PTEN expression in MRL/lpr mice compared with controls (Figure 5F), accompanied by decreased levels of Bcl‐2‐associated death promoter (BAD) and PRAS40 (Figure 5G,H). Silencing C5aR1 in vitro similarly increased PTEN expression, supporting a regulatory relationship between C5a/C5aR1 signaling and the PI3K/AKT pathway in LN.

## 4 DISCUSSION

This study revealed a key molecular mechanism by which the complement activation product C5a and C5aR1 promote the progression of LN by downregulating PTEN expression and activating the PI3K/AKT axis. Intervention with a C5aR1A resulted in significantly improved renal function and reduced tissue damage in LN mouse models. Collectively, these results not only deepen our understanding of LN pathogenesis but also establish the C5a/C5aR1–PTEN–PI3K/AKT axis as a critical pathogenic pathway and a promising therapeutic target for LNn.

C5a exhibits pathogenic mechanisms distinct from classical complement activation. The classical pathway is initiated by immune complexes via the C1q‐C3 complex, whereas the lectin pathway is triggered by mannose‐binding lectin (MBL).^22^ In contrast, C5a induces phosphorylation of the mitochondrial fission protein Drp1 at Ser616 through the calcium‐NFATc1 pathway, leading to mitochondrial dysfunction in podocytes.^23^, ^24^ This C5a‐specific effect supports a “complement hierarchy model,” in which C5a functions as an upstream regulator of inflammation, and the downstream membrane attack complex exerts pathogenic effects dependent on prior C5a activation.^25^ A positive feedback loop between C5a and MBL may further amplify renal inflammation.

This study suggests that targeting the C5a/C5aR axis may provide a more precise and safer therapeutic strategy than broad complement inhibition. Abnormal C5a/C5aR expression was closely associated with LN severity, indicating potential as a biomarker for disease activity.^24^ By modulating PTEN and inhibiting the PI3K/AKT pathway, this study indicates the potential to develop novel therapeutic agents to alleviate renal injury. The results further show that C5a‐induced PI3K/AKT hyperactivation promotes BAD phosphorylation and podocyte apoptosis, forming an inflammatory‐metabolic feedback loop.^26^ This mechanism may explain the limited efficacy of anti‐C3 therapies, as C3a shows a weak correlation with renal damage. Moreover, combined treatment with C5aRA and CTX produced better outcomes than either monotherapy, suggesting a synergistic effect that warrants further investigation.

This study proposes the following hypotheses regarding the regulation of PTEN. First, transcription factor regulation: multiple transcription factors can directly or indirectly modulate PTEN transcription. Under inflammatory or stress conditions, NF‐κB, p53, and Egr‐1 are involved in this process; inflammatory signaling can activate NF‐κB to suppress PTEN transcription, thereby enhancing PI3K/AKT activity and promoting inflammation and apoptosis.^27^ Second, non‐coding RNA regulation: miRNAs, such as miR‐21, regulate PTEN post‐transcriptionally by targeting PTEN mRNA for degradation or translational inhibition, reducing PTEN protein levels.^28^ Long non‐coding RNAs can also modulate PTEN indirectly by competitively binding miRNAs or influencing transcription factors.^29^ These regulatory mechanisms collectively contribute to PTEN dysfunction and PI3K/AKT hyperactivation. Combined with the observed negative regulation of PTEN by the C5a/C5aR axis in this study, it is proposed that this axis may cooperatively mediate PTEN downregulation through multiple pathways, particularly under inflammatory conditions characterized by signaling crosstalk and non‐coding RNA dysregulation, thereby promoting PI3K/AKT activation and the progression of LN.

Despite the robust experimental evidence obtained in the animal model, this study has several limitations. First, the current research focused primarily on the MRL/lpr mouse model, without clinical validation in SLE/LN patient samples. Consequently, the expression patterns of C5a, C5aR1, and PTEN in human LN tissues, as well as their correlation with disease severity and clinical outcomes, remain to be established. Second, although the MRL/lpr mouse is widely used in LN research, its pathology mainly reflects proliferative forms (e.g., Class IV) and may not fully capture the heterogeneity of human LN subtypes, such as Class V membranous lesions. This limits the generalizability of the findings. In addition, the upstream regulatory mechanisms controlling PTEN expression have not yet been fully elucidated. Future studies will require integration of multi‐omics approaches, including transcriptomics and proteomics, to comprehensively map the PTEN regulatory network.

To address these limitations, we propose the following directions for future research: Clinical validation: Expand the investigation to clinical cohorts by systematically evaluating the expression profiles of the C5a/C5aR1‐PTEN‐AKT axis across different LN subtypes, and analyzing their correlations with disease activity indices and renal pathology scores. This will help assess the axis's sensitivity and specificity as a diagnostic or prognostic biomarker. Therapeutic optimization: Assess the potential synergistic effects of combining C5aR1A with current immunosuppressive therapies, with the goal of developing more effective combination treatment strategies for LN. Mechanistic exploration: Investigate the upstream regulatory mechanisms of PTEN expression, with a focus on transcription factors, non‐coding RNAs, and epigenetic modifications. This may yield novel targets and strategies for precision intervention.

In summary, this study demonstrates that the C5a/C5aR1 axis promotes inflammation and tissue injury in LN by suppressing PTEN expression and activating the PI3K/AKT axis.^8^ Previous evidence has shown that C5aR1 signaling can activate the NF‐κB pathway, enhance proinflammatory cytokine production, and induce Th17 cell expansion, suggesting potential crosstalk with PI3K/AKT signaling in amplifying the inflammatory microenvironment.^12^ Moreover, C5aR1 plays a dual role in immune complex clearance and inflammation regulation, contributing both to proinflammatory responses and, under certain conditions, to immune resolution and tissue repair.^11^

## 5 CONCLUSION

This study elucidated the core mechanism by which the C5a/C5aR1 axis drives podocyte injury and renal dysfunction in LN through the downregulation of PTEN and subsequent activation of the PI3K/AKT pathway. Administration of a C5aR1A significantly suppressed AKT phosphorylation, restored PTEN expression, reduced proteinuria, and alleviated renal tissue damage in LN mouse models, suggesting the therapeutic potential of targeting the C5a/C5aR1‐PTEN axis. C5a expression levels were positively correlated with LN disease activity, indicating its potential utility as a noninvasive biomarker. Future efforts should focus on advancing the clinical translation of C5aR1A and exploring its combination with immunosuppressive agents to optimize subtype‐guided therapeutic strategies.

## AUTHOR CONTRIBUTIONS

Yuehong Ma and Xiaoshuang Zhou designed the research study. Yi Wang and Peng Zhao performed the experiments and data collection. Li Cheng and Lei Li analyzed the data and assisted with interpretation. Rongshan Li and Xiaoshuang Zhou supervised the project and revised the manuscript. Yuehong Ma drafted the initial manuscript. All authors reviewed and approved the final version of the manuscript.

## CONFLICT OF INTEREST STATEMENT

The authors declare no conflicts of interest.

## ETHICS STATEMENT

All animal experiments were approved by the Animal Ethics Committee of Shanxi Provincial People's Hospital (Approval No. 2021136).

## Supporting information

Supporting Information S1

## Data Availability

All data generated or analyzed during this study are included in this article and/or its Supporting Information S1 files. Further enquiries can be directed to the corresponding author.
