# Correction to Regulation of phosphatase and tensin homolog by complement component 5a (C5a) and its receptor (C5aR 1) in lupus nephritis: A novel therapeutic target

**DOI:** 10.1002/ccs3.70087

**Published:** 2026-06-25

Ma, Yuehong, YiWang, PengZhao, et al. 2025. “Regulation of Phosphatase and Tensin Homolog by Complement Component 5a (C5a) and Its Receptor (C5aR 1) in Lupus Nephritis: A Novel Therapeutic Target,” *Journal of Cell Communication and Signaling*: e70055. https://doi.org/10.1002/ccs3.70055.

In the originally published article, the first panel is missing from Figure 5e. The corrected figure is shown below. Additionally, throughout the article, “LNn” has been changed to “Ln” and “C5aR11” has been changed to “C5aR 1.” The online version of the article has been updated.

We apologize for this error.

**FIGURE 5:**
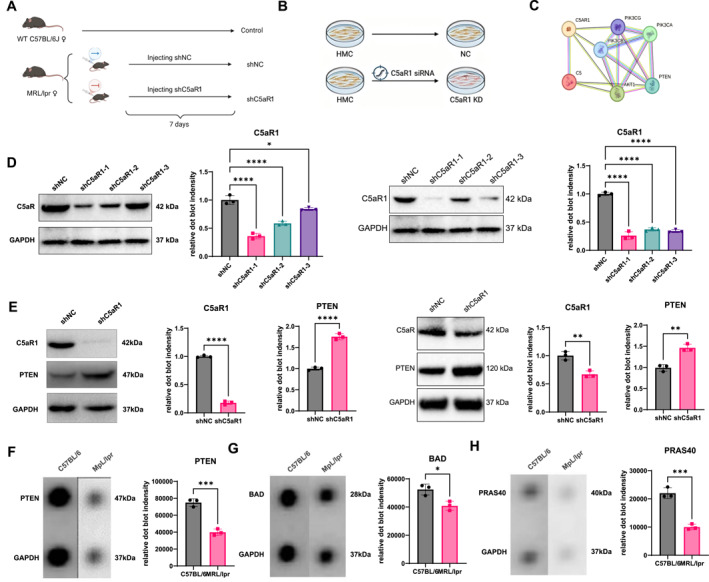
C5a suppresses PTEN expression and enhances AKT pathway activation to promote inflammation. (A) Schematic diagram of the animal model procedure; (B) Schematic diagram of the in vitro cell experiment; (C) Protein‐protein interaction network illustrating key molecules linking C5a/C5aR1 with PTEN and the PI3K/AKT signaling pathway (confidence score = 0.15); (D) Western blot analysis of C5aR1 knockdown by three siRNAs in vitro (D1–D2) and by three shRNAs in kidney tissues in vivo (D3–D4), with GAPDH as loading control (*n* = 3); (E) Western blot analysis of C5aR1 and PTEN expression after C5aR1 knockdown by siRNAs in vitro (E1–E3) and by shRNAs in kidney tissues in vivo (E4–E6), with GAPDH as loading control (*n* = 3). (F–H) Results of the human/mouse AKT pathway phosphorylation antibody array C1 (RayBiotech) showing the expression of BAD, PRAS40, and PTEN. Group comparisons were performed using a two‐tailed unpaired *t*‐test or one‐way ANOVA. *p >* 0.05, not significant; *p < 0.05; **p < 0.01; ***p < 0.001; ****p < 0.0001. ANOVA, analysis of variance; BAD, Bcl‐2‐associated death promoter; PTEN, phosphatase and tensin homolog. [Correction added on 21 May 2026, after first online publication: Figure 5 has been corrected in this version.]

