# Supplementary material for: Regulation of phosphatase and tensin homolog by complement component 5a (C5a) and its receptor (C5aR1) in lupus nephritis: A novel therapeutic target
Source: J Cell Commun Signal. 2025 Dec 19;19(4):e70055. doi: 10.1002/ccs3.70055 (PMC12716956; doi:10.1002/ccs3.70055)
Supplement: Supplementary file 1 — Supporting Information S1 [file CCS3-19-e70055-s001.docx]

**
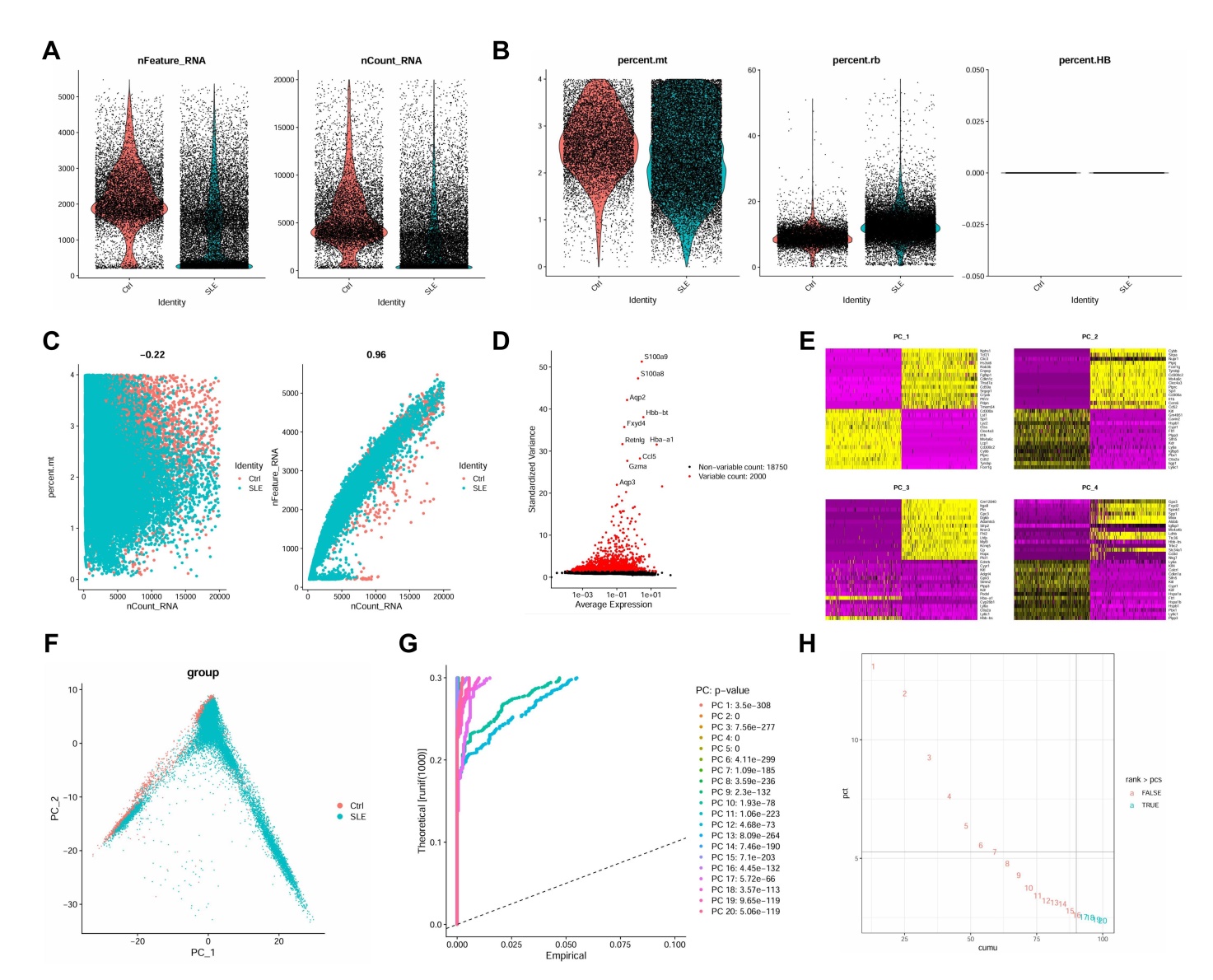
**

**Figure S1. Quality control and PC selection after filtering the dataset GSE279823.**

Note: (A) Violin plots of nFeature_RNA and nCount_RNA following data filtering; (B) Violin plots showing percent.mt, percent.rb, and percent.HB; (C) Correlation analysis of sequencing depth: nCount_RNA vs. percent.mt, and nCount_RNA vs. nFeature_RNA; (D) Scatter plot of gene expression variance, with red dots indicating highly variable genes used for PCA; (E) Heatmap of the top 30 genes associated with PC_1 to PC_4 in PCA, where yellow indicates upregulation and purple indicates downregulation; (F) Two-dimensional distribution of cells in PC_1 and PC_2; (G) JackStraw plot showing the *p*-values of PC_1 to PC_20 relative to a uniform distribution (dashed line), where significantly informative PCs are above the dashed line; (H) Elbow plot of standard deviation contribution across PCs, with the selected PCs for dimensionality reduction highlighted in orange.

**
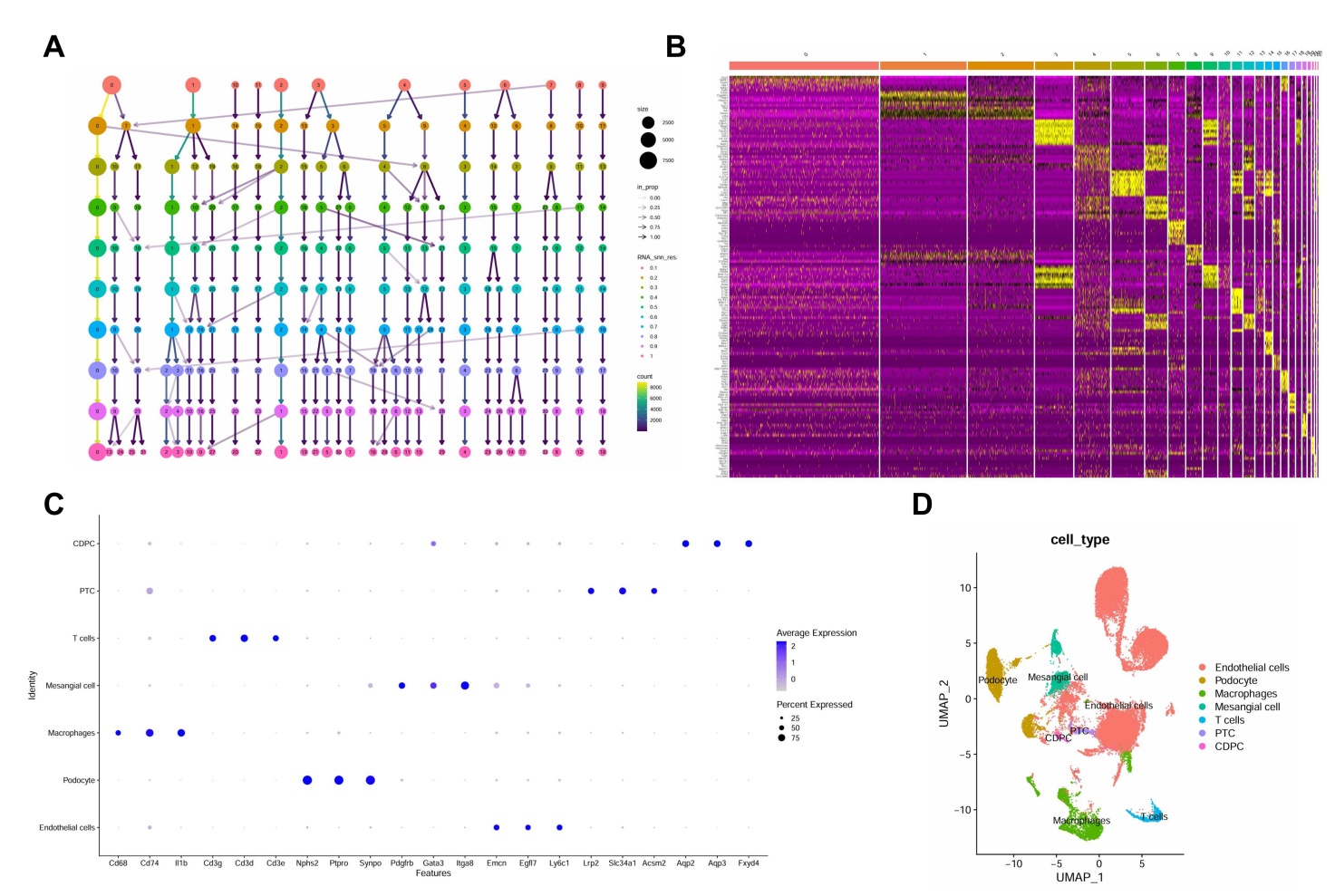
**

**Figure S2. Clustering analysis and selection of marker genes for dataset GSE279823.**

Note: (A) Visualization of clustering results under different resolution values (0.1-1) for dataset GSE279823; (B) Heatmap showing the top 10 marker genes for each cluster, where yellow indicates upregulation and purple indicates downregulation; (C) Dot plot of cell-type-specific marker genes used for annotation and their expression levels; (D) UMAP plot of annotated cell types in dataset GSE279823, with control and SLE groups combined.
